# New Insights into Immunopathology Associated to *Bothrops lanceolatus* Snake Envenomation: Focus on PLA_2_ Toxin

**DOI:** 10.3390/ijms24129931

**Published:** 2023-06-09

**Authors:** Joel J. M. Gabrili, Giselle Pidde, Fabio Carlos Magnoli, Rafael Marques-Porto, Isadora Maria Villas-Boas, Carla Cristina Squaiella-Baptistão, Felipe Silva-de-França, François Burgher, Joël Blomet, Denise V. Tambourgi

**Affiliations:** 1 Immunochemistry Laboratory, Butantan Institute, São Paulo 05503-900, Brazilgiselle.pidde@butantan.gov.br (G.P.); fabio.magnoli@butantan.gov.br (F.C.M.); isadora.boas@butantan.gov.br (I.M.V.-B.); carla.baptistao@butantan.gov.br (C.C.S.-B.); felipe.franca.esib@esib.butantan.gov.br (F.S.-d.-F.); 2 Development and Innovation Laboratory, Butantan Institute, São Paulo 05503-900, Brazil; rafael.porto@butantan.gov.br; 3 Prevor Laboratory, 95760 Valmondois, France; fburgher@prevor.com (F.B.); jblomet@prevor.com (J.B.)

**Keywords:** *Bothrops lanceolatus*, snake venom, PLA_2_, inflammation, complement system

## Abstract

The systemic increase in inflammatory mediator levels can induce diverse pathological disorders, including potentially thrombus formation, which may be lethal. Among the clinical conditions in which the formation of thrombi dictates the patient’s prognosis, envenomation by *Bothrops lanceolatus* should be emphasized, as it can evolve to stroke, myocardial infarction and pulmonary embolism. Despite their life-threatening potential, the immunopathological events and toxins involved in these reactions remain poorly explored. Therefore, in the present study, we examined the immunopathological events triggered by a PLA_2_ purified from *B. lanceolatus* venom, using an ex vivo human blood model of inflammation. Our results showed that the purified PLA_2_ from the venom of *B. lanceolatus* damages human erythrocytes in a dose dependent way. The cell injury was associated with a decrease in the levels of CD55 and CD59 complement regulators on the cell surface. Moreover, the generation of anaphylatoxins (C3a and C5a) and the soluble terminal complement complex (sTCC) indicates that human blood exposure to the toxin activates the complement system. Increased production of TNF-α, CXCL8, CCL2 and CCL5 followed complement activation. The venom PLA_2_ also triggered the generation of lipid mediators, as evidenced by the detected high levels of LTB_4_, PGE_2_ and TXB_2_. The scenario here observed of red blood cell damage, dysfunctions of the complement regulatory proteins, accompanied by an inflammatory mediator storm, suggests that *B. lanceolatus* venom PLA_2_ contributes to the thrombotic disorders present in the envenomed individuals.

## 1. Introduction

*B. lanceolatus*, also known as the Martinique lancehead (“Fer-de-Lance”) or the Martinique pit viper, is endemic to the island of Martinique. It is the only venomous snake species from this location, where it accounts for an average of 30 accidents annually [1]. Envenomed patients present the local and systemic reactions commonly reported for bothropic accidents [2,3], though with some peculiarities. At the venom inoculation site, a painful and intense edematogenic reaction is observed, which can affect the entire limb and the abdominal region. Some patients can also present local ecchymosis, necrosis and sometimes bleeding and blister formation [4,5]. The systemic reactions detected in these patients are characterized by vascular dysfunctions, such as hypo- and/or hypertension, and hemostatic disorders, such as thrombosis, observed in 30–40% of the patients [6,7]. This is unlike the clinical picture associated with the envenomation by the South American *Bothrops* species, which induces a consumptive coagulopathy evolving to multiple hemorrhage foci. These thrombotic reactions have been characterized as a Thrombotic Microangiopathy-like disease, with multiple tissue infarctions, evolving to stroke, heart attack and pulmonary embolism [8,9,10,11], which are the main causes of death resulting from these accidents.

Notably, the pathogenic events that lead to the thrombotic reactions in these accidents remain obscure because animal models do not reproduce human clinical symptoms [12]. Additionally, in vitro models using human samples of plasma and platelets did not reproduce coagulation activation events [1,6,9], suggesting that the hemostatic dysfunctions are mediated by additional elements, such as inflammatory reactions. In agreement with this idea, increased levels of acute phase proteins, such as C-reactive protein (CRP) and fibrinogen have been reported in patients envenomed by *B. lanceolatus* [6,10], suggesting that the inflammatory process contributes to the resulting pathology.

In addition, other inflammatory events were also detected in experimental systems in which human samples were evaluated. Our group [13] showed that the incubation of human serum with *B. lanceolatus* venom results in complement activation through the alternative, lectin and classical pathways, culminating in the generation of anaphylatoxins and sTCC. A part of this activation was due to the direct action of snake venom metalloproteases (SVMP) upon the central complement components (C3, C4, C5) and disruption of the inhibitory C1 inhibitor (C1-INH) properties. In particular, the direct cleavage of C5 by SVMPs culminated in the generation of the highly functional C5a anaphylatoxin, which triggered a robust Ca^2+^ signaling cascade in human neutrophils.

Recently, we have demonstrated [14], using the ex vivo human whole blood model, that the *B. lanceolatus* venom elicits a strong inflammatory mediator storm characterized by a high generation of complement activation products (C3a, C4a, C5a, sTCC), lipid mediators (LTB_4_, PGE_2_, TXB_2_), interleukins (IL-1β, IL-6, TNF-α) and chemokines (CCL2, CCL5, CXCL8). Interestingly, Guimaraes et al. [15] showed that the pretreatment of the venom with EDTA, a Ca^2+^ chelating agent, led to the inhibition of local inflammatory reactions in rats, suggesting that Ca^2+^ dependent venom toxins were the main immunopathological inducers. PLA_2_ is among the venom toxins that require Ca^2+^ for their catalytic activity [16].

PLA_2_ (EC 3.1.1.4) consists of an enzyme group that is very important for a multitude of physiological, immunological and pathological processes. These enzymes hydrolyze glycerophospholipids in the sn-2 position, releasing lysophospholipids and free fatty acids [17,18,19]. PLA_2_ enzymes are classified into sixteen groups, which are clustered in six main classes. Among them, the secreted PLA_2_ (sPLA_2_) class can be highlighted due to its ubiquitous presence in snake venoms, as well as to its role in diverse clinical conditions [19]. The snake venom sPLA_2s_ present Mr of 14–18 kDa, with five to eight disulfide bridges and a His/Asp catalytic dyad in their active site that requires Ca^2+^ for its enzymatic activity.

Snake venom PLA_2_s exhibits diverse envenoming toxic activities including the ability to promote coagulation disturbances [20], pain [21], tissue damage [22], neurotoxicity [23] and death [24]. These different toxic effects are closely associated to the various functional sites on the surface of PLA_2_s and their diverse binding receptors [25].

Thus, considering that these enzymes are broadly found in *Bothrops* snake venoms [26], including *B. lanceolatus* [12,27], and that these proteins elicit a large range of molecular effects in immune [28] and non-immune cells [29,30], it is possible that their action can contribute to the prothrombotic events resulting from envenomation by *B. lanceolatus*. Therefore, in this study, we have investigated the immunopathological actions of a PLA_2_ purified from *B. lanceolatus* venom, using the ex vivo human whole blood model.

## 2. Results

### 2.1. PLA_2_ Isolation from the B. lanceolatus Venom

**Figure 1:**
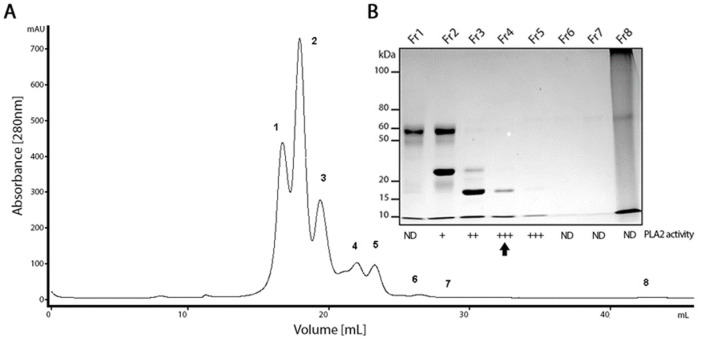
Purification of phospholipase A2. (**A**) Chromatogram of the *B. lanceolatus* venom (20 mg) fractionated by size-exclusion chromatography in an FPLC system. (**B**) Silver stained SDS-PAGE of the 8 fractions (Fr1 to Fr8) obtained, and detection of PLA_2_ (PLA_2_ activity). (+) 5 to 39 UF/min/µg; (++) 40 to 99 UF/min/µg; (+++) ≥ 100 UF/min/µg; (ND) not detected. Arrow highlights fraction 4 which contains a band of 15 kDa and a high PLA_2_ activity.

**Figure 2:**
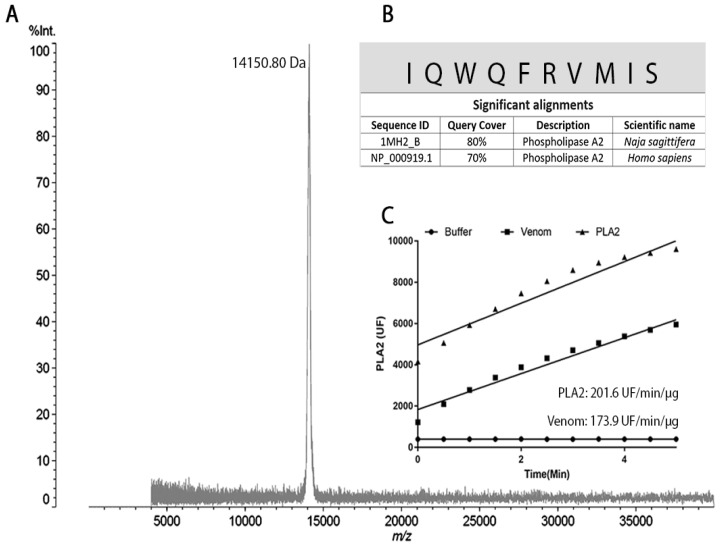
Identification of phospholipase A_2_. (**A**) MALD-TOF MS analysis of fraction 4. (**B**) N-terminal sequence of the first 10 amino acid residues of the purified PLA_2_ determined by automatic Edman degradation, showing a high sequence homology with PLA_2_ from *Naja sagittifera* (80%) and *H. sapiens* (70%). (**C**) Determination of the specific PLA_2_ activity of the purified protein (Fr4) compared to the crude venom.

The crude venom of *B. lanceolatus* was fractionated by size-exclusion chromatography, resulting in eight chromatographic fractions (Figure 1A). All fractions were tested for PLA_2_ activity using a fluorimetric assay. Fractions two to five were positive (Figure 1B). Fraction four showed a higher PLA_2_ activity and a single protein band of approximately 15 kDa in SDS-PAGE (Figure 1B and Figure 2C).

MALDI-TOF-MS analysis of peak four confirmed that the fraction contained a single molecule with a molecular mass of 14,150.80 Da (Figure 2A). The N-terminal sequence of the protein was determined by automatic Edman degradation, resulting in the identification of ten amino acids, which showed high sequence homology with a PLA_2_ from *Naja sagittifera* (80%) and a PLA_2_ from *H. sapiens* (70%) (Figure 2B).

Fluorometric analysis showed that the purified protein presents a PLA_2_-specific activity of 201.6 UF/min/µg and the crude venom of *B. lanceolatus* (positive control) has a specific activity of 173.9 UF/min/µg (Figure 2C).

### 2.2. B. lanceolatus Venom PLA_2_ and Hemoglobin Release

**Figure 3:**
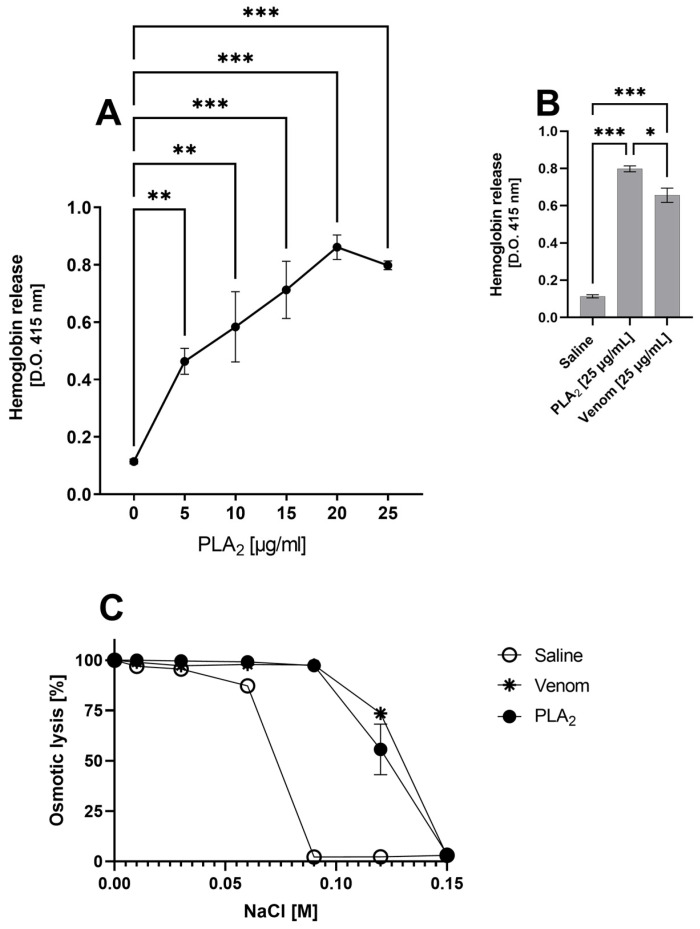
Hemolysis and osmotic fragility of human erythrocytes induced by *B. lanceolatus* venom PLA_2_. (**A**,**B**) Hemoglobin release after incubation with increasing concentrations of PLA_2_ (5, 10, 15, 20 and 25 μg/mL), crude venom (25 μg/mL) or saline (negative control) for 30 min at 37 °C. After incubation, the absorbance of the plasma was measured at 415 nm. (**C**) Human whole blood was treated with PLA_2_ (5 μg/mL), *B. lanceolatus* venom (25 μg/mL) or saline (0.15 M) for 30 min at 37 °C. Erythrocytes were then exposed to decreasing concentrations of NaCl (0.15, 0.12, 0.09, 0.06, 0.03, 0.01 and 0). After incubation for 30 min at 37 °C, non-lysed cells were pelleted; the absorbance of the supernatant was measured at 415 nm and expressed as percentage of lysis. Results are representative of three different experiments carried out in duplicate and represented as mean ± SD. * *p* ≤ 0.05, ** *p* ≤ 0.01, *** *p* ≤ 0.001 (one-way ANOVA followed by Tukey post-test).

The *B. lanceolatus* purified PLA_2_ was tested for its hemolytic activity using human blood. Figure 3 shows a dose dependent release of hemoglobin promoted by *B. lanceolatus* PLA_2_ (panel A), and also, the activity of the whole venom (panel B). Figure 3C also shows that the purified PLA_2_, as well as the whole *B. lanceolatus* venom, increases susceptibility of human erythrocytes to osmotic lysis when compared to the cells treated with saline (negative control) (Figure 3C). It is important to mention that we have selected the lowest concentration of PLA_2_, able to promote direct hemolysis (Figure 3A: 5 mg), to perform the next experiments of the present study. This PLA_2_ hemolytic dose is also in line with data from the literature [31].

### 2.3. B. lanceolatus Venom PLA_2_ Activates the Complement System in the Ex Vivo Human Whole Blood Model

We have previously demonstrated that *B. lanceolatus* venom’s serino- and metalloproteases activate the complement system in human serum. To verify whether PLA_2_ can also interfere with the complement system, we incubated the purified toxin or buffer alone as the negative control with human whole blood samples for 30 min.

**Figure 4:**
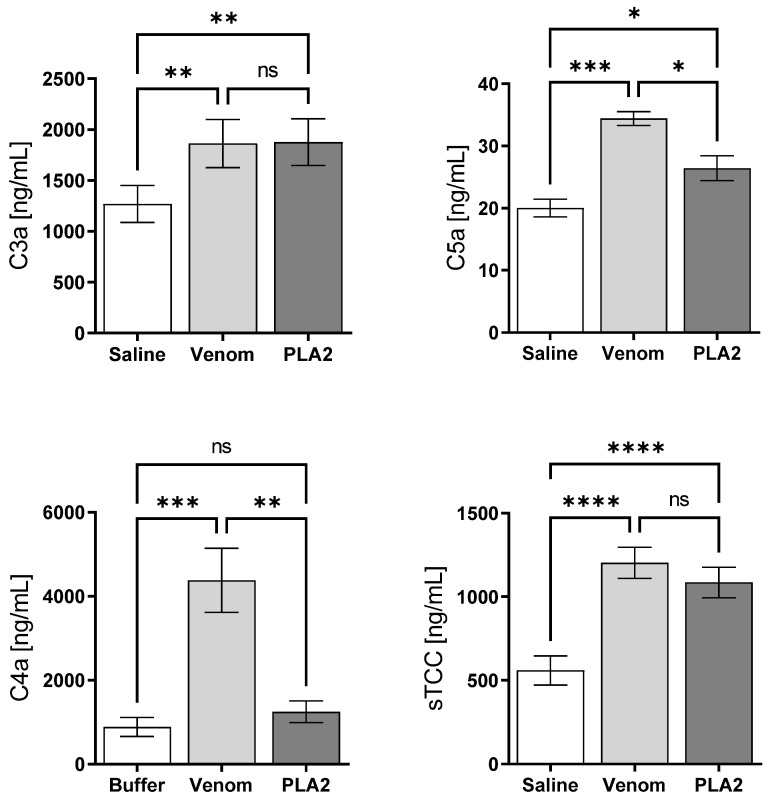
Generation of anaphylatoxins and sTCC in whole human blood stimulated with *B. lanceolatus* venom PLA_2_. Samples of human blood were incubated with *B. lanceolatus* venom (25 μg/mL), PLA_2_ (5 μg/mL) or saline for 30 min. After collection and dilution of the plasma (1:1000), production of C3a/C3a-desArg, C4a/C4a-desArg and C5a/C5a-desArg anaphylatoxins was measured by the Cytometric Bead Array. Production of sTCC was measured using the MicroVue SC5b-9 Plus EIA Kit. Results are represented as mean ± SD of the duplicates. Data are representative of three independent experiments. * *p* ≤ 0.05, ** *p* ≤ 0.01, *** *p* ≤ 0.001, **** *p* ≤ 0.0001 (one-way ANOVA followed by Tukey post-test). ns: non significative difference.

Figure 4 shows that PLA_2_, like the whole venom, is able to activate the complement system, as revealed by the presence of C3a/C3a desArg, and C5a/C5a desArg and sTCC in the plasma samples. However, only the whole venom was also able to promote the generation of C4a/C4a desArg (Figure 4).

### 2.4. B. lanceolatus Venom PLA_2_ Induces Modulation of Erythrocyte Membrane Complement Regulators

**Figure 5:**
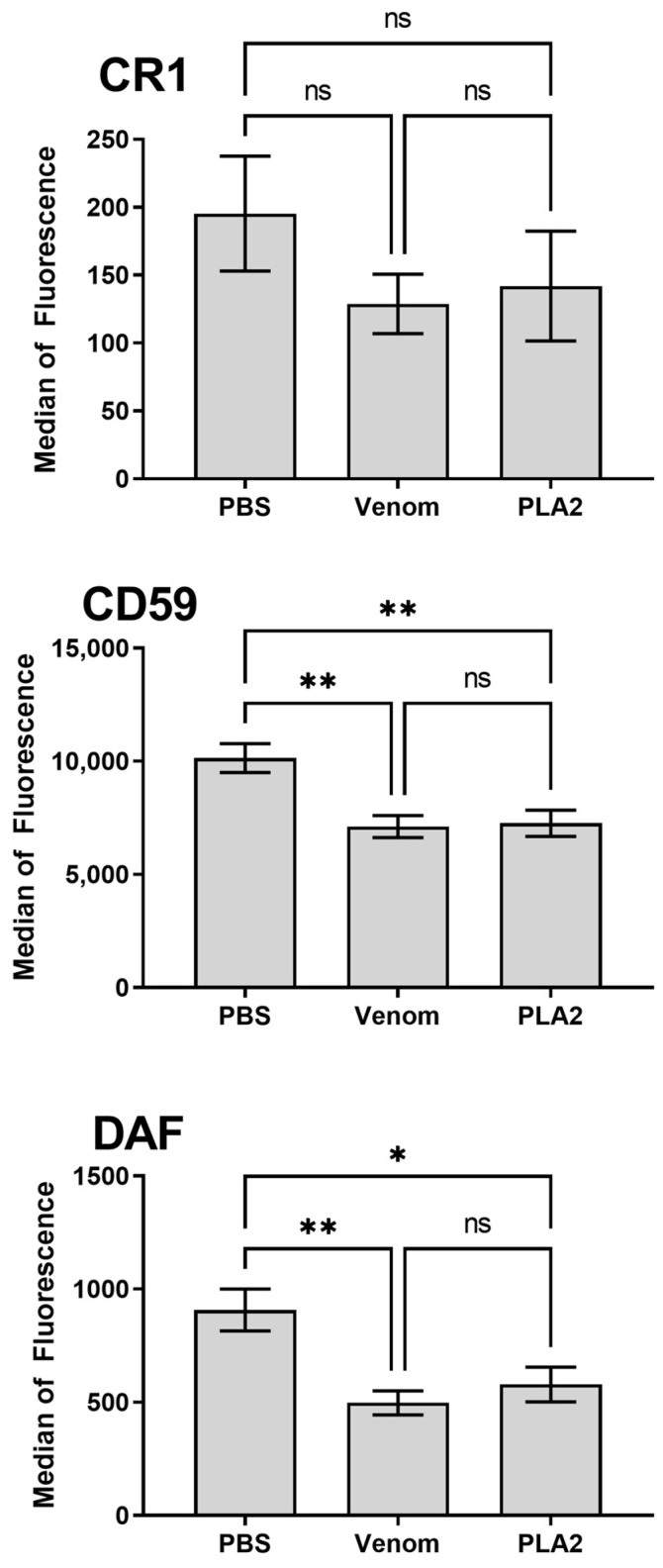
Erythrocyte complement regulators levels decrease upon treatment with the *B. lanceolatus* venom PLA_2_. Whole blood was treated with *B. lanceolatus* venom (25 μg/mL), PLA_2_ (5 μg/mL) or saline for 30 min, and erythrocytes were then analyzed for the detection of CR1, DAF and CD59. Results are representative of three different experiments and expressed as the mean of triplicates ± SD. * *p* ≤ 0.05, ** *p* ≤ 0.01 (one-way ANOVA followed by Tukey post-test). ns: non significative difference.

To assess whether PLA_2_ could also affect the erythrocytes’ complement membrane regulators, cells were analyzed for the presence of the Decay accelerating factor (DAF; CD55), Complement receptor 1 (CR1; CD35) and MAC-inhibitory protein (CD59) by flow cytometry. Both PLA_2_ and venom promoted a significant reduction in the detection of CD59 and DAF, as compared to the control cells treated with buffer, after 30 min of treatment. Nonetheless, detection of CR1 did not change upon treatment with the venom or PLA_2_ (Figure 5).

### 2.5. B. lanceolatus Venom PLA_2_ Induces Pro-Inflammatory Cytokines and Chemokines in Human Whole Blood

**Figure 6:**
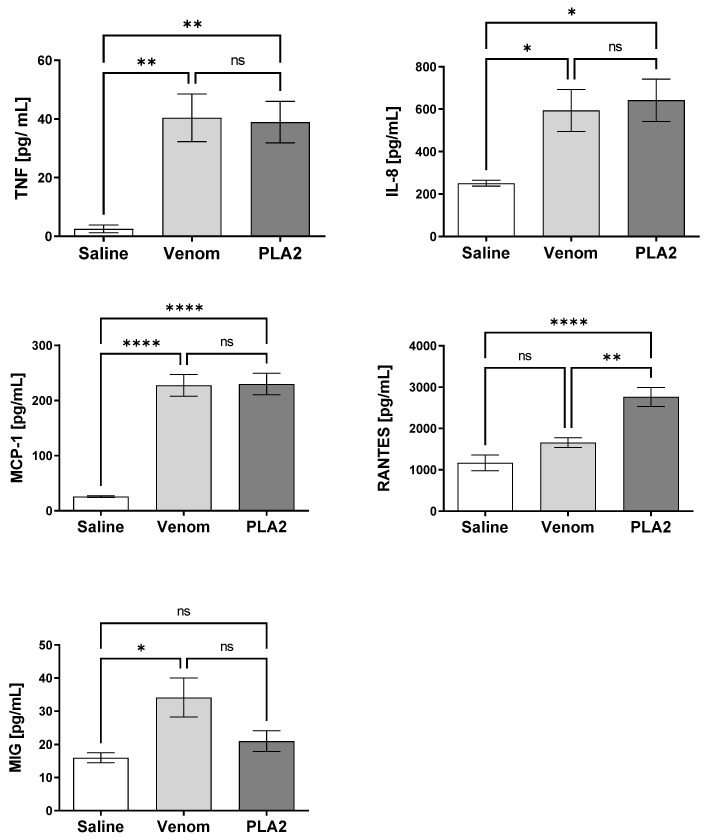
Levels of cytokines and chemokines in whole human blood stimulated with *B. lanceolatus* venom. Samples of human blood were incubated with *B. lanceolatus* venom (25 μg/mL) or PLA_2_ (5 μg/mL) or saline for 30 min. The production of cytokines and chemokines was determined in the separated plasma by the Cytometric Bead Array. Results are presented as mean ± SD of the triplicates. Data are representative of three independent experiments. * *p* ≤ 0.05, ** *p* ≤ 0.01, **** *p* ≤ 0.0001 (one-way ANOVA followed by Tukey post-test). ns: non significative difference.

Pro-inflammatory cytokine and chemokine production, induced by the *B. lanceolatus* venom and PLA_2_, was analyzed in human blood after 30 min of incubation. PLA_2_ induced the production of TNF, CXCL8/IL-8, CCL2/MCP-1 and CCL5/RANTES. *B. lanceolatus* venom could also induce the production of TNF, CXCL8/IL-8 and CCL2/MCP-1, but differently from the isolated PLA_2_, it also induced an increase in the generation of CXCL9/MIG, but not of CCL5/RANTES (Figure 6).

### 2.6. B. lanceolatus Venom Induces the Release of Lipid Mediators

**Figure 7:**
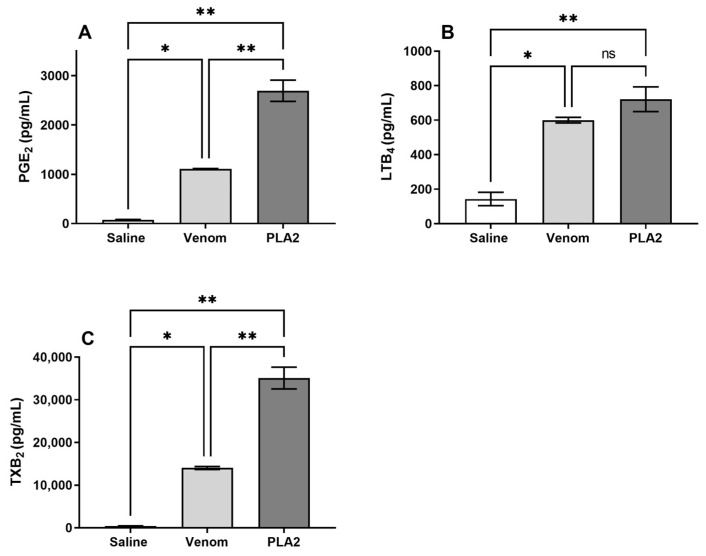
Lipid mediator production is elicited by *B. lanceolatus* venom and its PLA_2_. Human whole-blood samples were incubated with *B. lanceolatus* venom (25 µg/mL), PLA_2_ (5 µg/mL) or with saline for 30 min, and PGE_2_ (**A**), LTB_4_ (**B**) and TXB_2_ (**C**) were quantified by ELISA. Data are representative of three independent experiments. * *p* ≤ 0.05, ** *p* ≤ 0.01 (one-way ANOVA followed by Tukey post-test). ns: non significative difference.

Bioactive lipids, including PGE_2_, LTB_4_ and large amounts of TXB_2_, a stable TXA_2_ metabolite, were detected in human whole blood after incubation with *B. lanceolatus* venom or the isolated PLA_2_. Interestingly, the treatment with PLA_2_ promoted a production of higher levels of PGE_2_ and TXB_2_ than the treatment with whole *B. lanceolatus* venom (Figure 7).

## 3. Discussion

Envenomation by *B. lanceolatus* presents a clinical picture characterized by a systemic thrombotic syndrome and important local inflammation, but limited hemorrhage, which differs from the hemorrhagic syndrome caused by South American bothropic envenomations. *B. lanceolatus* venom cleaves purified human fibrinogen, but is unable to clot citrated human plasma, and an almost normal coagulation profile can be observed in patients developing thrombosis [32]. The fact that *B. lanceolatus* venom does not alter the coagulation profile in vitro prompted us to test it in a whole human blood model, using lepirudin as a blood anticoagulant, which does not interfere with the complement cascade activation.

Here, we aimed to evaluate the immunopathological actions of a PLA_2_ isolated from *B. lanceolatus* venom in a human whole blood model. Purified PLA_2_ activated the complement system, as evidenced by the presence of sTCC in whole blood plasma samples, as well as of C3a/C3a desArg and C5a/C5a desArg, but not C4a/C4a desArg. These data suggest that PLA_2_ activates the alternative but not the classical or lectin pathways. On the contrary, whole venom promotes the generation of C4a/C4a desArg, suggesting that other venom components can activate the classical and/or the lectin pathways.

Complement activation was accompanied by the release of lipid mediators, which included LTB_4_, PGE_2_ and TXB_2_. In addition, in blood exposed to PLA_2_, we detected the production of TNF, CXCL8/IL-8, CCL2/MCP-1 and CCL5/RANTES, which shows that PLA_2_ isolated from *B. lanceolatus* venom causes a strong inflammatory reaction in the blood, establishing favorable conditions for thrombus formation. Curiously, blood exposure to the toxin triggered a strong release of the CCL5 chemokine and the TXB_2_ prostanoid, which points to platelet activation since both mediators are classically characterized as activation markers of this cell type [33]. Notably, we previously detected that the crude venom of *B. lanceolatus* induces the same effect [14]. We therefore hypothesized that the thrombotic reactions observed in human envenomation by this snake was in part mediated by platelet activation. This could take place through complement activation, since studies have shown that the inhibition of C5a Receptor 1 (C5aR1) signaling reduces the generation of TXB_2_ in human whole blood samples exposed to the *Naja annulifera* (snouted/banded cobra) venom [34].

We also observed a decrease in the levels of the complement regulators DAF and CD59 on the erythrocyte membrane. The best-known function of DAF is regulating the complement system activity by inhibiting C3 (C4b2a and C3bBb) and C5 convertases (C4b2a3b and C3bBb3b) on the cell surface [35]. CD59 is the main cell surface inhibitor of the complement membrane attack complex (MAC: a multimolecular complex composed of C5, C6, C7, C8 and C9n), which is formed as a consequence of complement activation. Reduction in the CD55 and CD59 expression levels is observed in some autoinflammatory diseases and immunodeficiency disorders in which a decrease in complement regulator expression on the cell surface contributes to complement mediated-hemolysis, systemic low-grade inflammation, thrombosis, microangiopathic reactions and renal failure [36]. Thus, the imbalance on CD55 and CD59 expression, and the assembly of significant levels of sTCC after human whole blood exposure to PLA_2_ could lead red blood cells to destruction. This pathological event should be further investigated since it may be an envenomation signature for some species of venomous animals. 

Indeed, exposition of human blood to *Trimeresurus flavoviridis* snake venom results in sTCC formation, reduction of CD55 and CD59 molecules on the erythrocytes’ cell membrane, and in hemolysis [37,38]. In addition, the venoms from *Loxosceles* spiders and its major toxin, the Sphingomyelinase D (SMase D), induce the activation of endogenous cell membrane metalloproteases on human erythrocytes, which in turn cleave Glycophorins A, B and C, transforming these cells into autologous complement activators, with hemolysis as the final event [39]. Glycophorins are molecules rich in sialic acid, an alternative complement pathway regulator. Thus, the decrease in their expression transforms the erythrocytes susceptible to complement attack, leading the individual to develop disseminated intravascular hemolysis, which is a pathological signature of systemic loxosceslim [39] 

Strikingly, the exposure of the human whole blood to the *B. lanceolatus* PLA_2_ toxin culminated in C3a and C5a anaphylatoxin generation, while C4a was not detected, which suggests that a part of the immunopathological reactions described here could be mediated by the complement alternative pathway activation as observed in *Loxosceles* envenoming [39]. 

Interestingly, diseases in which CD55 and CD59 expression is dysregulated, the patients present increased levels of molecular markers of the alternative pathway activation, so, these markers should be examined in future studies, since they can be used, for instance, as envenomation prognostic biomarkers and as possible therapeutic targets.

The decrease in CD55 and CD59 erythrocyte cell surface expression detected here can be partially responsible for complement activation. However, a plethora of molecular mechanisms in this envenomation can cooperate to protect or potentiate the organism’s imbalance caused by *B. lanceolatus* crude venom and its purified toxins. Indeed, in previous studies of our group, we detected an increased expression in CD59 and CD46 complement regulators on endothelial cell membranes induced by *B. lanceolatus* venom [13]. CD46 is a cell bound complement regulator, which is also capable of triggering TCD4+ and CD8+ cell activation, promoting their effector functions [40,41,42,43]. Thus, considering that (i) different classes of inflammatory mediators were detected here and by others [11], (ii) high circulating *B. lanceolatus* venom levels are observed in envenomed individuals [44] and (iii) increased levels in CD46 within the endothelium could in turn activate these cells, it is possible to consider that endothelial cells contribute to the thrombotic events of *B. lanceolatus* envenomation [11]. Therefore, the endothelial cells’ influence on the thrombotic disorders associated with *B. lanceolatus* envenomation should be minutely investigated.

Interestingly, the purified toxin induces a dose dependent lysis of human erythrocytes, which may be related to an increased osmotic fragility of these cell membranes. On the other hand, a decrease in membrane C-regulators in PLA_2_-treated erythrocytes can make these cells susceptible to complement attack, which would provide an additional mechanism of hemolysis.

During the hemolysis process, hemoglobin (Hb) is released from erythrocytes into the circulation and triggers the production of different Hb redox states and heme, which can act as Damage-Associated Molecular Patterns (DAMPs). Recently, it has been shown that heme can have a direct action in hemolysis-associated complement activation. In fact, heme has been shown to activate the complement alternative pathway and trigger the deposition of C3 activation fragments on the surface of red blood cells [45]. Additionally, activation of the complement alternative pathway was detected on heme-exposed endothelial cells, resulting in C3 cell binding and MAC formation, a mechanism that contributes to endothelial damage and thrombosis in the atypical hemolytic uremic syndrome [36]. Thus, extracellular heme, by acting in both inflammatory and hemostatic pathways, can trigger a thromboinflammatory cycle that may contribute to the pathogenesis of hemolytic diseases.

In conclusion, the data presented here show that *B. lanceolatus* venom PLA_2_ activates the complement alternative pathway and induces a strong inflammatory scenario with the production of cytokines, chemokines and lipid mediators. The mechanisms involved in complement activation can be triggered either by a direct action of PLA_2_ on complement cascades or by an indirect action, i.e., induction of hemolysis, release of hemoglobin and activation of the complement system. Moreover, because the activity of PLA_2_ decreases the levels of C-regulators DAF and CD59 on the erythrocyte cell membrane, its hemolytic effect may be potentiated. This can lead to an increased susceptibility to complement lysis. Thus, our data suggest that PLA_2_s and complement activation are important factors in the genesis of the thrombotic events resulting from envenomation by *B. lanceolatus*.

## 4. Materials and Methods

### 4.1. Chemicals and Reagents

Bovine serum albumin (BSA) and ethylene diamine tetracetic acid (EDTA) were purchased from Sigma (St. Louis, MO, USA). Anticoagulant lepirudin (Refludan^®^) was from Celgene (Summit, NJ, USA). Sodium azide was from Vetec Química Fina LTDA (Rio de Janeiro, RJ, Brazil). BD Cytometric Bead Array (CBA) kits for Human anaphylatoxins, cytokines and chemokines were from BD Biosciences (San Jose, CA, USA). BCA kit “Protein Assay Kit” was purchased from Thermo Fisher Scientific (Schaumburg, IL, USA). The sTCC measurement was performed with the MicroVue SC5b-9 Plus EIA kit obtained from QUIDEL (San Diego, CA, USA).

### 4.2. Venom

Freeze-dried venom from *Bothrops lanceolatus* (*B. lanceolatus*) was obtained from Latoxan (Aix-en-Provence, France). Stock solutions were prepared in sterile saline solution at 5 mg/mL and stored at −80 °C. By using the BCA Protein Assay Kit (Thermo Fisher Scientific, Schaumburg, IL, USA), the protein concentration of the reconstituted venom was determined, according to the manufacturer’s instructions.

### 4.3. PLA_2_ Purification

Twenty milligrams of freeze-dried *B. lanceolatus* venom was fractionated by size exclusion chromatography (SEC) on a Superdex 200 10/300 GL column (GE Healthcare Bio-Sciences AB, Uppsala, Sweden). The column was equilibrated with ammonium acetate 50 mM pH-7.5, same as the running buffer. SEC was performed at room temperature using an ÄKTA Purifier 10 plus system under a 24 mL/h flow rate, and the absorbance at 280 nm of the eluate was monitored. The protein content of the obtained fractions was evaluated using the BCA Protein Assay Kit. The fractions were analyzed by SDS-PAGE [46] in a 12% gel, and silver-stained [47] in order to assess their protein complexity and size range. Fractions were then pooled and assayed for their phospholipase activity.

### 4.4. MALDI-TOF MS

MALDI-TOF MS analyses were performed in an AXIMA Performance MALDI TOF/TOF Mass Spectrometer (Shimadzu, Nakagyo-ku, Kyoto, Japan). Briefly, 0.5 µL of the sample was co-crystalized with an equal volume of sinapinic acid matrix solution and the mixture was spotted and dried on a steel target plate. Samples were analyzed in positive, linear mode under a vacuum of 3 × 10^−7^ Torr and laser power of 120 in a 4 kDa to 40,000 kDa window. 

### 4.5. N-Terminal Sequencing

N-terminal sequencing was performed by automated Edman degradation in a PPSQ-21A Protein Sequencer (Shimadzu, Kyoto, Japan). The dried samples were dissolved in 30 µL of TFA 0.1% and spotted on a fiberglass membrane. The membrane was dried, and the sample was sequenced following the manufacturer’s instructions and protocols. The spectra were generated by averages of 50–100 automatic laser shots. The mass spectra were analyzed using the Axima Performance proteomics suit.

### 4.6. Phospholipase A_2_ Activity

The PLA_2_ activity of the purified protein from the *B. lanceolatus* venom (5 µg) was analyzed using EnzChek^®^ Phospholipase A_2_ Assay Kit (Invitrogen, Eugene, OR, USA) following the manufacturer’s instructions. Saline and *B. lanceolatus* venom were used as negative and positive controls, respectively. Fluorescence measurements were performed at 37 °C using the spectrometer FLUOstar Omega (BMG Labtech, Offenburg, Germany) at the wavelength λEM = 515 nm, with an excitation of λEX = 485 nm every 30 s for 5 min. Specific activity was expressed as units of free fluorescence of cleaved substrate/min/μg of protein.

### 4.7. Hemolysis Assay

Human blood samples from healthy donors were collected in an anticoagulant (Alsever old solution: 114 mmol/L citrate, 27 mmol/L glucose, 72 mmol/L NaCl, pH 6.1) to obtain erythrocytes for subsequent use as target cells. The cells were washed and resuspended at saline and incubated with increasing concentrations of PLA_2_ (5, 10, 15, 20 and 25 μg/mL) or crude venom (25 μg/mL). Control samples were incubated with saline. After incubation for 30 min at 37 °C, unlysed cells were spun down; the absorbance of the supernatant was measured at 415 nm. Mean and SD were determined from duplicate samples.

### 4.8. Human Whole Blood Model

We used the human whole blood model described by Mollnes et al. [48]. Blood samples from healthy consenting donors were collected by venipuncture into tubes containing 50 µg/mL lepirudin (Refludan, Celgene, NJ, USA), the recombinant form of hirudin, a thrombin-inhibitor anticoagulant that does not interfere with the complement cascade. Immediately after collection, blood samples were transferred to 15 mL falcon tubes (Corning Inc., New York, NY, USA) (72% of total volume, *v*/*v*) and incubated with saline, *B. lanceolatus* venom (25 µg/mL) or purified PLA_2_ (5 µg/mL) (28% of the total volume, *v*/*v*) for 30 min in a water bath at 37 °C under agitation. The tubes were then centrifuged at 404× *g* at 4 °C for 10 min to collect the plasma. The plasmas were aliquoted and stored at −80 °C in the presence of EDTA (10 mM) for further analysis. Cells were prepared for osmotic susceptibility assay and for flow cytometry, as described below.

### 4.9. Osmotic Susceptibility

Erythrocytes obtained from the whole blood model, as described above, were washed and resuspended at 2% in saline. Cells were distributed in 96-well U-bottom plates (100 µL/well), centrifuged and resuspended in 100 µL of different NaCl concentrations (0, 0.01, 0.03, 0.06, 0.09, 0.12 and 0.015 M). After 30 min of incubation at 37 °C, the samples were centrifuged, and the absorbance was determined at 415 nm to measure the hemoglobin release. The intensity of hemolysis was expressed in percentage and plotted against the NaCl concentration in order to construct an osmotic fragility curve.

### 4.10. Detection of Anaphylatoxins and Soluble Terminal Complement Complex (sTCC) in Venom/Toxin Treated Samples

The generation of the anaphylatoxins (C3a/C3a-desArg, C4a/C4a-desArg and C5a/C5a-desArg) was determined using the Human Anaphylatoxin Cytometric Bead Array-CBA (BD Biosciences Pharmingen, San Diego, CA, USA), following the manufacturer’s instructions. Cytometric analysis was performed using a FACSCanto II (Becton Dickinson, San Diego, CA, USA), and the data were analyzed using the Flow Cytometric Analysis Program (FCAP) Array 3.0 (Becton Dickinson, San Diego, CA, USA). Anaphylatoxins concentrations (µg/mL) were determined by linear regression from the standard curve. The generation of sTCC (SC5b-9) was determined using the MicroVue SC5b-9 Plus EIA Kit (Quidel Corporation, San Diego, CA, USA), according to the manufacturer’s instructions. The concentration of sTCC (µg/mL) in the samples was calculated from a linear regression of the standard curve.

### 4.11. Analysis of Complement Regulators on Erythrocytes by Flow Cytometry

Erythrocytes obtained from the whole blood model, as described above, were distributed in FACS tubes (25 µL/tube) and incubated for 30 min at 4 °C with mouse monoclonal antibodies against CD59 [Bric 229 (IBGRL, Bristol, UK) diluted 1:250], DAF [Bric 216 (IBGRL, Bristol, UK) diluted 1:100], or with rabbit serum anti-CR1 (Santa Cruz Biotechnology Inc., Santa Cruz, CA, USA; diluted 1:50) diluted in FACS buffer. Next, the cells were washed and incubated for 30 min more with the secondary antibodies, i.e., anti-mouse FITC (Sigma, St. Louis, MI, USA) or anti-rabbit Alexa 488 (Thermo Fisher Scientific, Schaumburg, IL, USA) for 30 min at 4 °C. Cells were washed and fixed in FACS buffer containing 1% paraformaldehyde and analyzed by flow cytometry (FACSCanto II, Becton Dickinson, San Jose, CA, USA), using the software BD FACSDiva, version 4.1 (BD Biosciences, Franklin Lakes, NJ, USA), and data were expressed as median fluorescence intensity (MFI) ± SD.

### 4.12. Dosage of Cytokines and Chemokines in Human Whole Blood

The presence of the cytokines (IL-2, IL-4, IL-6, IL-10, IL-17A, IFN-γ and TNF) and chemokines (CXCL8/IL-8, CCL5/RANTES, CXCL9/MIG and CCL2/MCP-1) in plasma samples collected from human whole blood assays was assessed using “Cytometric Bead Arrays” (CBA): “Human Inflammatory Cytokine Kit”, “Human Th1/Th2/Th17 Kit” and “Human Chemokine Kit” (BD Biosciences, Franklin Lakes, NJ, USA), following the manufacturer’s instructions. The amount of each cytokine or chemokine was calculated (pg/mL of plasma) using the linear regression curve deduced from the standard curve.

### 4.13. Quantification of Lipid Mediators

Leukotriene B_4_ (LTB_4_), Prostaglandin E_2_ (PGE_2_) and Thromboxane B_2_ (TXB_2_) were quantified by the LTB_4_ ELISA, PGE_2_ Monoclonal ELISA and TXB_2_ ELISA kits, respectively, all from Cayman Chemical (Ann Arbor, MI, USA).

### 4.14. Statistical Analysis

Data were analyzed by one-way ANOVA followed by Tukey post-test using GraphPad Prism software v.9.5 (GraphPad Software, La Jolla, CA, USA). The differences were considered significant at *p* < 0.05.

## Data Availability

Not applicable.
